# Cranitomy vs. Forceps

**Published:** 1869-05-15

**Authors:** 


### *Craniotomy vs. Forceps.*

In a recent discussion upon this subject, before the St. Louis Medical Society, Dr. Boisliniere characterized craniotomy as "a relic of barbarian midwifery," asserting that it was demonstrated by statistics to be almost as fatal to the mother as the Cæsarian Section (the proportion of fatal cases in the former being 1 to $2\frac{3}{4}$, and in the latter 1 to 3, without any hope for the child). An analysis of the opinions apparently the most diverse and opposite demonstrated a growing sentiment in favor of the more frequent and earlier use of the forceps.

